# Manipulating the Lewis antigen specificity of the cholesterol-dependent cytolysin lectinolysin

**DOI:** 10.3389/fimmu.2012.00330

**Published:** 2012-11-05

**Authors:** Sara L. Lawrence, Susanne C. Feil, Jessica K. Holien, Michael J. Kuiper, Larissa Doughty, Olan Dolezal, Terrence D. Mulhern, Rodney K. Tweten, Michael W. Parker

**Affiliations:** ^1^Biota Structural Biology Laboratory and Australian Cancer Research Foundation Rational Drug Discovery Centre, St. Vincent’s Institute of Medical ResearchFitzroy, VIC, Australia; ^2^Victorian Life Sciences Computation Initiative, The University of MelbourneParkville, VIC, Australia; ^3^CSIRO Materials Science and EngineeringParkville, VIC, Australia; ^4^Department of Biochemistry and Molecular Biology, Bio21 Molecular Science and Biotechnology Institute, The University of MelbourneParkville, VIC, Australia; ^5^Department of Microbiology and Immunology, University of Oklahoma Health Sciences CenterOklahoma City, OK, USA

**Keywords:** cholesterol-dependent cytolysins, Lewis antigens, molecular dynamics simulations, protein engineering, surface plasmon resonance, X-ray crystallography

## Abstract

The cholesterol-dependent cytolysins (CDCs) attack cells by punching large holes in their membranes. Lectinolysin from *Streptococcus mitis* is unique among CDCs due to the presence of an N-terminal lectin domain that enhances the pore-forming activity of the toxin. We recently determined the crystal structures of the lectin domain in complex with various glycans. These structures revealed the molecular basis for the Lewis antigen specificity of the toxin. Based on this information we have used *in silico* molecular modeling to design a mutant toxin, which we predicted would increase its specificity for Lewis y, an antigen found on the surface of cancer cells. Surprisingly, we found by surface plasmon resonance binding experiments that the resultant mutant lectin domain exhibited higher specificity for Lewis b antigens instead. We then undertook comparative crystallographic and molecular dynamics simulation studies of the wild-type and mutant lectin domains to understand the molecular basis for the disparity between the theoretical and experimental results. The crystallographic results revealed that the net number of interactions between Lewis y and wild-type versus mutant was unchanged whereas there was a loss of a hydrogen bond between mutant and Lewis b compared to wild-type. In contrast, the molecular dynamics studies revealed that the Lewis b antigen spent more time in the binding pocket of the mutant compared to wild-type and the reverse was true for Lewis y. The results of these simulation studies are consistent with the conclusions drawn from the surface plasmon resonance studies. This work is part of a program to engineer lectinolysin so that it will target and kill specific cells in human diseases.

## INTRODUCTION

Lectinolysin (LLY) is a pore-forming toxin derived from some strains of *Streptococcus mitis* and *S. pseudopneumoniae* and is a member of the cholesterol-dependent cytolysin (CDC) family (Farrand et al., 2008). These pore-forming bacterial toxins are secreted as soluble monomers that assemble on the surface of cholesterol-rich cell membranes. The resultant pores are composed in excess of 30 monomers, and are greater than 150 Å in diameter (Hotze and Tweten, 2012). Perforation of the membrane by these pores results in cell lysis. The sequence similarity is high (40–80%) between CDC family members.

*Streptococcus mitis* can cause diseases such as infective endocarditis and septicemia (Hall and Baddour, 2002; Huang et al., 2002; Gowda et al., 2003; Kennedy et al., 2004). Serum isolates from Kawasaki disease patients were found to contain *S. mitis* human platelet aggregation factor (PAF or Sm-hPAF), so named because it was found to affect light scattering properties of human platelets, thought to be as a consequence of platelet aggregation (Ohkuni et al., 1997). Farrand et al. (2008) found that the Sm-hPAF gene sequence encodes a predicted CDC structure (Rossjohn et al., 1997; Polekhina et al., 2005), most closely related to intermedilysin (Farrand et al., 2008). Having established that PAF was in fact a member of the CDC family, it was renamed lectinolysin, because of its ability to bind carbohydrate (see below).

While LLY shares a number of characteristics typical of CDCs, it has a unique 162 amino acid N-terminal domain (LLY^lec^; Farrand et al., 2008). Sequence comparisons show that the LLY^lec^ domain shares significant identity with fucose-binding proteins. Glycan array experiments revealed that LLY was highly selective for the difucosylated glycans Lewis y (Le^y^) and Lewis b (Le^b^) antigens (Farrand et al., 2008). Small-angle X-ray scattering (SAXS) modeling predicts that LLY^lec^ is joined to CDC domain 1 so that the Le antigen-binding site is exposed on the outer surface of the pore (Feil et al., 2012).

Lewis antigens are blood group determinants with very rigid structures (Yuriev et al., 2005). Le^b^ is a type 1 antigen and adsorbed onto the surface of blood cells. Le^y^ is a type 2 antigen which, in healthy individuals, is expressed at low levels in tissues including epithelial cells and serum (Yuriev et al., 2005; Westwood et al., 2009). However, expression is elevated on the surface of a wide range of epithelial tumor cells including colon, lung, and ovary (Sakamoto et al., 1986; Miyake et al., 1992; Yin et al., 1996). Thus, Le^y^ is a distinctive tumor marker and therefore a promising target for directing drugs to cancer cells.

Various approaches have been taken to produce Le^y^-specific cancer therapeutics, including the development of Le^y^-specific humanized monoclonal antibodies (Kitamura et al., 1994; Clarke et al., 2000a,b; Scott et al., 2000), and anti Le^y^ T cells (Westwood et al., 2005). A chimeric approach has also been taken whereby a Le^y^-specific monoclonal antibody was conjugated to the CDC, listeriolysin O, to form an immunotoxin (Bergelt et al., 2009). LLY is a naturally occurring, potential oncotoxin, with in-built tumor targeting specificity for the Le^y^ antigen. However, cross reactivity with Le^b^ antigen decreases its usefulness as a specific, cancer-targeting molecule.

Previously we have determined the crystal structure of LLY^lec^, and its complexes with fucose and Le^b^ and Le^y^ antigens (Feil et al., 2012). The overall fold of the LLY^lec^ domain exhibited similarities with fucolectin domains of the CBM family 98 glycoside hydrolase of *Streptococcus pneumonia* and *Anguilla anguilla* agglutinin. The binding of Le^y^ and Le^b^ to LLY^lec^ was very similar with the exception of an additional hydrogen bond between Le^b^ and amino acid residue Tyr62 (Feil et al., 2012).

Using these structures, we have explored the possibility of mutating LLY^lec^ to develop a Le^y^-specific binding domain. In the work presented here a Y62H mutation was designed and constructed with the aim of removing the hydrogen bond between the *N*-acetylglucosamine (Glc*N*Ac) of Le^b^ and LLY^lec^ and instead, gain a hydrogen bond with Le^y^. However, binding studies described here show that LLY^lec^Y62H has a greater affinity for Le^b^ over Le^y^, whereas LLY^lec^wt has a preference for Le^y^ over Le^b^. In order to explore the molecular basis for this unexpected finding we determined the crystal structure of LLY^lec^Y62H in complex with Le^b^ and Le^y^. We then compared these structures with those of the wild-type lectin domain complexed with the same Lewis antigens. The crystallographic studies were complemented by molecular dynamics simulations in order to explore the importance of protein motion and interaction with the ligands over time.

## MATERIALS AND METHODS

### *IN SILICO* DESIGN OF MUTANTS

The crystal structures of the lectin domain of LLY in complex with Le^b^ antigen (PDB code: 3LEK) and Le^y^ antigen (PDB code: 3LEG) were visually inspected using Pymol^1^. *In silico* mutations were created and assessed also using the Pymol program. Figures were created in Pymol and using Chemaxon software^2^.

### SURFACE PLASMON RESONANCE BIOSENSOR BINDING ANALYSIS

All surface plasmon resonance (SPR) experiments were performed at 25°C using a Biacore T200 instrument (GE Healthcare). Direct assays were performed with Le^y^ and Le^b^ antigens (Sigma) injected over mutant and wild-type LLY^lec^ domain proteins immobilized on a CM5 chip (GE Healthcare). Immobilizations were performed in 1 × HBS-P running buffer [10 mM HEPES, 150 mM NaCl, 0.005% (w/v) Tween 20]. Binding experiments were performed in 1 × HBS-P + running buffer [10 mM HEPES, 150 mM NaCl, 0.05% (w/v) Tween 20] containing 1 mM MgCl_2_, 1 mM CaCl_2_, and 0.2 mg/ml bovine serum albumin.

LLY^lec^wt and LLY^lec^Y62H proteins were immobilized in two separate channels on a CM5 chip using a standard amine coupling protocol. Briefly, the chip surface was activated with a single 5 min injection of freshly prepared 1:1 50 mM *N*-hydroxysuccinimide:200 mM 3-(*N,N*-dimethylamino)propyl-*N*-ethylcarbodiimide. Protein coupling was achieved by three 5 min injections of LLY^lec^wt or LLY^lec^Y62H solution (50 μg/ml in 10 mM sodium acetate, pH 5.0). To deactivate residual reactive sites, lectin domain coupling was followed by a 5 min injection of 1 M ethanolamine (pH 8.5). Approximately 3000 response units (RU; 1 RU = 1 pg of protein/mm^2^) of LLY^lec^wt (channel 2) and 4000 RU of LLY^lec^Y62H (channel 3) were coupled. Channel 1 was activated and blocked, as above, for use as a reference surface.

Le^b^ and Le^y^ were injected, in duplicate, in a twofold dilution series from 1 mM to 7.5 μM over the immobilized wild-type and mutant lectin domains. Thirty-second injections of Le antigen at 40 μl/min were followed by a 60-s dissociation period. Baseline returned to 0 almost immediately after the association phase ended, so regeneration of the surface was not required.

Binding data were processed and analyzed using Scrubber 2 software^3^ (version 2.0c). Rapid association and dissociation rates made data fitting to a kinetic model and subsequent calculation of kinetic rate constants *k*_a_ and *k*_d_ impractical. Consequently, equilibrium dissociation constants (*K*_D_) were derived by fitting binding responses at equilibrium to a 1:1 steady-state affinity model available within Scrubber.

### MUTAGENESIS, EXPRESSION, AND PURIFICATION OF THE LLY^lec^ DOMAINS

A mutant of LLY^lec^, LLY^lec^Q190C, with an N-terminal 6 × His tag and a TEV protease cleavage site for His-tag removal, was used in this study. The LLY^lec^Q190C coding region corresponds to LLY residues 38–190 (GenBank accession number AB051299.1). The C-terminal residue was mutated to cysteine (Q190C) for protein labeling studies. The point mutant displays wild-type activity and is located far from the Lewis antigen-binding site (Farrand et al., 2008). Henceforth, LLY^lec^Q190C will be described as LLY^lec^wt in this work. Expression was carried out in *E. coli* BL21, and the protein purified using nickel resin and size exclusion chromatography as previously described (Feil et al., 2012). LLY^lec^wt was mutated with QuikChange^TM^ Site-Directed Mutagenesis Kit (Stratagene) to create the Lewis antigen-binding site mutant LLY^lec^Y62H. LLY^lec^wt and LLY^lec^Y62H were purified to >95% purity, as determined by SDS gel electrophoresis. Proteins were concentrated to 10 mg/ml in 10 mM Tris–HCl pH 7.2, 10 mM NaCl, and 5 mM dithiothreitol, and were stored at -80°C.

### CRYSTALLIZATION OF THE LLY^lec^Y62H MUTANT

All crystallizations were performed using the hanging drop vapor diffusion method at 21°C. 2 μl of protein was mixed with equal volume of precipitant and hung over 0.5 ml of well solution. LLY^lec^Y62H crystallization conditions were determined by fine screening around the conditions that proved successful for the wild-type protein (Feil et al., 2012): 2 M MgSO_4_ and 100 mM Tris–HCl buffer pH 8.2–9.0. Optimization included the addition of the Hampton additive screen (Hampton Research, CA, USA) 10% (v/v) in the hanging drops. The optimal crystallization conditions were 2.4 M MgSO_4_ and 100 mM KCl with 100 mM Tris–HCl pH 8.75–9.0. Le^y^ and Le^b^ antigens were soaked into LLY^lec^Y62H crystals by adding 1 μl 1 M MgSO_4_, 100 mM Tris–HCl pH 9.0, 100 mM KCl, 5 mM Le^y^ or Le^b^ to the crystal drop for 1 h at 21°C. Soaks were carried out immediately prior to cryoprotection and flash freezing in liquid nitrogen. Crystals were cryoprotected for X-ray data collection by adding glycerol in increments of 5% (v/v) to a final concentration of 20% (v/v) to the crystal drops.

### CRYSTALLOGRAPHIC STUDIES OF THE LLY^lec^Y62H MUTANT

Diffraction data were collected at the MX2 beamline at the Australian Synchrotron in Clayton, Victoria. The data collection was controlled using Blue-Ice software (McPhillips et al., 2002). The diffraction data were processed using the HKL2000 suite (Otwinowski and Minor, 1997). Model building was performed with COOT (Emsley and Cowtan, 2004) using the published wild-type crystal structure of LLY^lec^ (PDB code: 1LE0) as a starting model. Data were refined with REFMAC 5 (Murshudov et al., 1997) from the CCP4 program suite (CCP4, 1994). Restrained positional and isotropic temperature factor refinement was employed until convergence and the refinement was monitored using the *R*_free_ residual. The Lewis antigens were fully occupied in the binding sites of both structures and their temperature factors were very similar to those of the surrounding side-chains.

### MOLECULAR MODELING OF THE LLY^lec^ INTERACTION WITH THE Le^b^ AND Le^y^ ANTIGENS

To investigate the mechanism of association and disassociation between LLY^lec^ and Lewis antigens four molecular models were used: two being the published crystal structures of LLY^lec^wt bound to either Le^b^ or Le^y^ antigen (Feil et al., 2012) and the other two being the crystal structures of LLY^lec^Y62H, complexed to the same ligands, that are described here. The molecular dynamics program NAMD (Phillips et al., 2005) was used to model ligand disassociation of each complex with a 30-ns simulation from the initial starting bound conformation, repeated 100 times for a cumulative simulation of 3 μs for each system. Models were initially solvated with TIP3 water under periodic boundary conditions of dimensions 48 Å × 48 Å × 64 Å. Charges were neutralized with NaCl for a total ionic concentration of 150 mM. The bound calcium ion seen in the crystal structures was included in the simulations. Dynamic molecular modeling was conducted at a theoretical pH of 7.4. Each simulation run started with an equilibration phase of 0.5 ns, where the protein backbone and Lewis antigen was harmonically constrained to their starting positions at 310 K and an NPT ensemble (an isothermal and isobaric ensemble where the number of moles (N), pressure (P), and temperature (T) are conserved). Subsequent 30-ns production simulations were run with no constraints at 310 K under NVT ensembles [a canonical ensemble where the number of moles (N), volume (V), and temperature (T) are conserved]. Trajectory snapshots were captured every 100 ps. At the completion of 100 repeat simulations, all production runs per model were consolidated into a single trajectory and the frames root-mean-square (rms) deviation were centered, based on the LLY alpha carbon protein backbone. The Lewis antigen was then subject to clustering analysis, whereby the top 20 ligand conformational clusters with a cut-off of 1.5 Å were generated and averaged. Ligand clusters with a rms deviation value less than 3.5 Å from the initial position were considered to be in a bound conformation.

## RESULTS

### *IN SILICO* DESIGN OF MUTANTS

**FIGURE 1:**
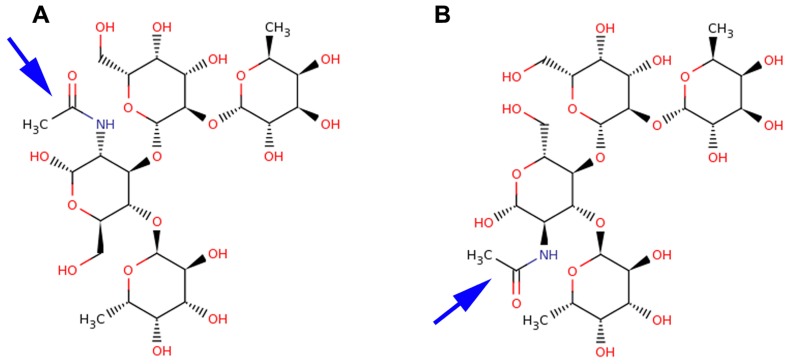
**Structural comparison of Le^b^ and Le^y^ antigens**. Lewis antigens Le^b^ **(A)** and Le^y^ **(B)** are structurally very similar. They differ in the core disaccharide linkages and the opposing projections of their *N*-acetyl (blue arrow) and CH_2_OH groups of the Glc*N*Ac moiety.

The difference between Le^y^ and Le^b^ antigens is the core disaccharide linkage (Galβ1-4 versus Galβ1-3) with an opposing projection of the *N*-acetyl glucosamine (Glc*N*Ac) *N*-acetyl and –CH_2_OH groups of the *N*-acetylglucosamine monosaccharide as shown in **Figure 1**. The overall Lewis determinants maintain the same conformation in both free and bound states.

**FIGURE 2:**
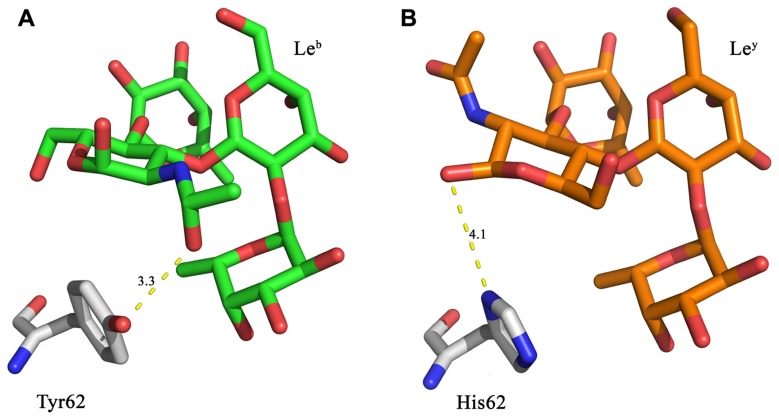
***In silico* LLY^lec^Y62H mutant design**. **(A)** Le^b^ is within hydrogen bonding distance (3.3 Å) of Tyr62 from the LLY^lec^wt domain. **(B)** A Y62H mutation was designed to retain similar structural properties to tyrosine, with the possibility that it may also result in a hydrogen bond to the Le^y^ antigen. Yellow dashed lines denote hydrogen bonds.

In the crystal structure of LLY^lec^wt the Glc*N*Ac of Le^b^ is within hydrogen bonding distance (3.3 Å) of Tyr62 (**Figure 2A**; Feil et al., 2012). This interaction is not able to occur with Le^y^, thus suggesting that mutating Tyr62 may alter the affinity for Le^b^. Various mutations of this residue were created *in silico*. Mutation to a histidine residue would maintain structural similarity of the binding site whilst also creating the potential for a new interaction between the histidine side-chain and Le^y^ as illustrated in **Figure 2B**. Replacement of Tyr62 with His was expected to remove the interaction between Le^b^ and the hydroxyl group of Tyr62 (**Figure 2A**).

### SPR BIOSENSOR BINDING ANALYSIS

**FIGURE 3:**
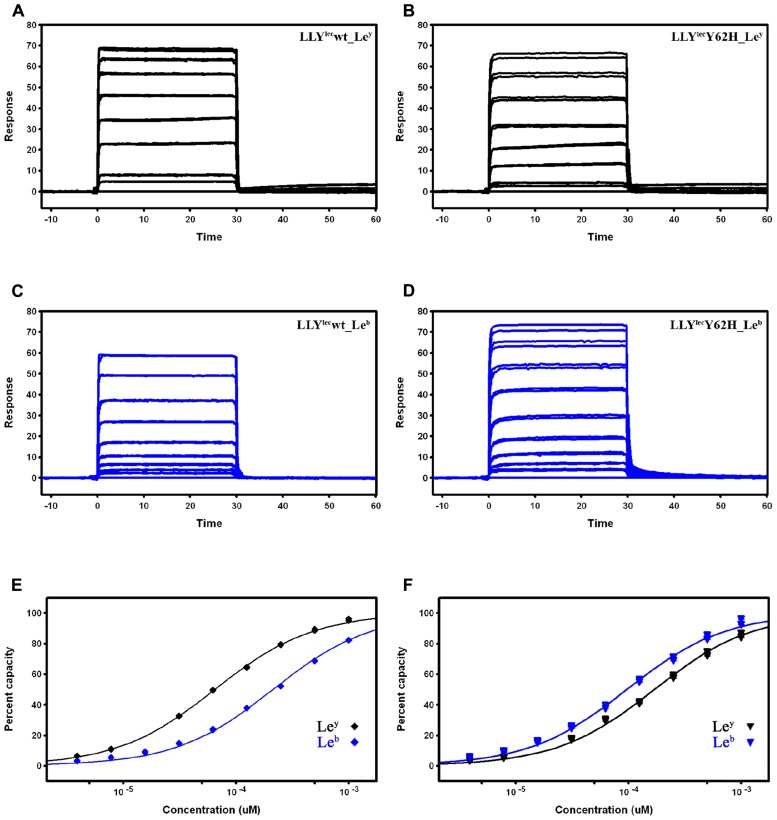
**SPR measurements of immobilized LLY^lec^wt and LLY^lec^Y62H**. Le^y^ antigen and Le^b^ antigen were injected at concentrations of 7.6–1000 μM. **(A)** LLY^lec^wt vs Le^y^, **(B)** LLY^lec^Y62H vs Le^y^, **(C)** LLY^lec^wt vs Le^b^, **(D)** LLY^lec^Y62H vs Le^b^. Responses at equilibrium fitted to simple 1:1 binding isotherms for Le^y^ (black lines) and Le^b^ (blue lines) interacting with **(E)** LLY^lec^wt and **(F)** LLY^lec^Y62H mutant. Duplicate binding data sets are shown.

Purified LLY^lec^Y62H yield was 20 mg/l *E. coli* BL21 culture in Luria–Bertani (LB) broth. This yield was equivalent to that found for LLY^lec^wt. The sensorgrams from SPR experiments between the Lewis antigens (Le^b^ and Le^y^) and either wt or LLY^lec^Y62H are shown in **Figure 3**. The estimated *K*_D_ values are listed in **Table 1**. The data indicate low affinity binding interactions of the Lewis antigens with the lectin domains. The wild-type lectin domain (LLY^lec^wt) has more than threefold greater affinity for Le^y^ over Le^b^. The LLY^lec^Y62H mutant has a 1.6-fold greater preference for Le^b^ over Le^y^. The mutation causes an overall 2.5-fold decrease in the lectin domain’s affinity for Le^y^ and doubles its affinity for Le^b^ over the wild-type domain. Overall, the data show that wild-type LLY^lec^ preferentially binds Le^y^ antigen while the mutant LLY^lec^Y62H exhibits higher affinity for Le^b^ than for Le^y^.

**Table 1:** Estimated equilibrium dissociation constants (*K*_D_) for LLY^lec^Y62H and LLY^lec^wt domains interacting with Le^y^ and Le^b^ antigens.

	LLY^lec^wt *K*_D_ (μM)	LLY^lec^Y62H *K*_D_ (μM)
Le^y^	78 ± 9	202 ± 31
Le^b^	234 ± 12	127 ± 14

### CRYSTAL STRUCTURES OF LLY^lec^Y62H IN COMPLEX WITH Le^y^ AND Le^b^ ANTIGENS

LLY^lec^Y62H crystallization was optimized in 100 mM Tris pH 8.75–9.0, 2.4 M MgSO_4_, and 100 mM KCl. Small bipyramidal crystals of dimensions 0.15 mm × 0.12 mm × 0.12 mm appeared in 6–8 weeks. After 8 months some crystals had grown to 0.25 mm × 0.2 mm × 0.2 mm. In contrast, LLY^lec^ wt crystals appeared after 5 days in 100 mM Tris pH 8.2–8.4, 2.0 M MgSO_4_ with the same morphology as LLY^lec^Y62H, but reach the same size within 10 days (Feil et al., 2012). Lewis antigens were soaked into crystals immediately prior to cryoprotection and freezing.

The crystal structures of the LLY^lec^Y62H in complex with the Lewis antigens was performed as described in Materials and Methods. The crystal structures have been deposited in the PDB with codes: 4GWJ and 4GWI for the Lewis b and Lewis y complexes, respectively. Data statistics for the structures are listed in **Table 2**.

**Table 2:** Crystallographic data processing and refinement statistics for LLY^lec^wt and LLY^lec^Y62H crystal structures in complex with Le^y^ and Le^b^ antigens.

	LLY^lec^Y62H-Le^y^	LLY^lec^Y62H-Le^b^
**Data collection**		
Space group	*P4*_3_*2*_1_*2*	*P4*_3_*2*_1_*2*
Unit cell dimensions (Å)	67.1, 67.1, 99.4	66.9, 66.9, 99.4
Wavelength (Å)	0.95	0.95
Temperature (K)	100	100
Maximum resolution (Å)	1.6	1.6
No. of observations	426,761	404,032
No. of unique reflections	30,563	9,510
Redundancy	14.0	13.7
Data completeness (%)	99.2 (93.7)	96.7 (79.3)
I/σI	19.6 (7.1)	16.9 (5.3)
*R*merge (%)A	11.5 (45.8)	10.9 (57.1)
**Refinement**		
Non-hydrogen atoms		
Protein	1124	1103
Water	146	150
Mg^2+^	2	3
Ca^2+^	1	1
Ligands	46	46
Resolution (Å)	1.6	1.6
*R_work_* (%)^B^	16.9	18.0
*R_free_* (%)	19.1	21.4
Rms deviations from ideal geometry		
Bond lengths (Å)	0.028	0.028
Bond angles (θ)	1.4	1.4
Bonded B’s	3.9	4.1
Mean B (Å^2^)		
Main-chain	22.8	27.0
Side-chain	28.2	31.9
Water	36.9	29.4
Ligand	53.5	43.8
Residues observed	41 to 184	43 to 184
Residues in most favored regions of the Ramachandran plot (%)	87.7	88.4
Residues in the disallowed regions of the Ramachandran plot (%)	0	0

*The values in parentheses are for the highest resolution bin (approximately 0.1 Å width)*. ^A^*R_merge_* = Σ_*hkl*_ Σ*_i_|I_i_* – *<I>|/|<I>|, where I_i_ is the intensity for the ith measurement of a symmetry related reflection with indices h,k,l*. ^B^*R_work_*. = Σ*||F_obs_|*-*|F_calc_||/*Σ*|F_obs_|, where F_obs_ and F_calc_ are the observed and calculated structure factor amplitudes, respectively.*

LLY^lec^ adopts an eight-stranded β-sandwich fold, composed of a five-stranded anti-parallel β-sheet on one side and a three-stranded anti-parallel β-sheet on the other side. Three short α-helices separate β-strands 1 and 2 and 3 and 4. There is one calcium ion in the structure that is in the same position as observed in structurally related fucolectin domains. There is also a metal ion that is covalently bound to His80, as well as five water molecules, all arranged in octahedral geometry. In our previous work we identified the ion as either a Ni^2^^+^ or Mg^2^^+^ ion (Feil et al., 2012) and here we have chosen the latter possibility. The Le^y^ and Le^b^ antigen-binding site is in a cleft at one end of the molecule. A more detailed description of the structure has been reported elsewhere (Feil et al., 2012). In the new mutant structures some additional N-terminal residues are observed (three extra in the Le^y^ structure and one extra in the Le^b^ structure), compared to the published wild-type structures (see **Table 2**).

**FIGURE 4:**
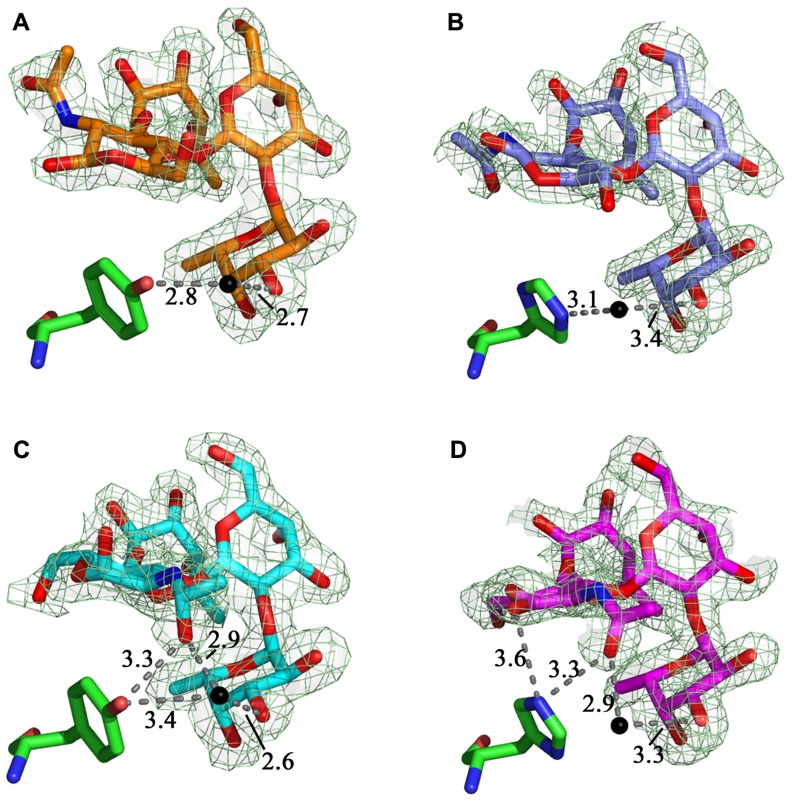
**LLY^lec^wt and LLY^lec^Y62H** domains in complex with Lewis antigens. Comparison between **(A)** the LLY^lec^wt-Le^y^ complex (orange carbons, green Tyr62) and **(B)** the LLY^lec^Y62H-Le^y^ complex (purple carbons, green His62) shows that the water molecule shifts toward the histidine residue in LLY^lec^Y62H-Le^y^. The water-mediated hydrogen bond to the hydroxyl group of Tyr62 seen in the LLY^lec^wt-Le^b^ complex (cyan carbons, green Tyr62) (**C**) was lost in the LLY^lec^wt-Le^b^ structure (magenta carbons, green Tyr62) (**D**) as this residue was replaced by His62. Hydrogen bonds are shown in gray and water molecules as black spheres. The fit of the ligands to the relevant 2*F*_o_–*F*_c_ electron density map (dark green hash), contoured at 1σ, are shown.

The structures of the LLY^lec^wt-Le^y^ (**Figure 4A**) and LLY^lec^Y62H-Le^y^ (**Figure 4B**) superimpose very closely, with a rms deviation of the alpha carbon atoms of 0.1 Å. A third metal ion binding site is observed in the mutant structure and we have tentatively identified it as a Mg^2^^+^ ion binding site due to the very high concentrations of this ion in the crystallization buffer. The Mg^2^^+^ ion is bound between the carboxylate of Asp97 and the side-chain carbonyl of Gln54 and four water molecules, all arranged in octahedral geometry around the metal ion. The water structure is the same as in LLY^lec^wt-Le^y^ apart from a water molecule that is positioned between the hydroxyl group of Tyr62 and fucose 1 (Fuc 1; **Figure 4A**). This water molecule is shifted by 1.4 Å toward the NE2 of His62 in the mutant structure (**Figure 4B**), compared to wild-type (**Figure 4A**), to optimize its interaction with the His side-chain. LLY^lec^Y62H forms the same 19 van der Waals interactions with Le^y^ compared to the wild-type protein: 15 with Fuc 1, 1 with Gal, 2 with Glc*N*Ac, and 1 with Fuc 2. The B-factor of Le^y^ is 53.5 Å^2^ in the mutant (overall B-factor of protein is 29 Å^2^), compared to 38.2 Å^2^ in the wild-type protein (overall B-factor of protein complex is 21 Å^2^). Overall, there is no net change in the number of potential hydrogen binding and van der Waals interactions between wild-type and mutant Le^y^ complexes.

Superposition of the structures of the LLY^lec^wt-Le^b^ and LLY^lec^Y62H-Le^b^ gives a rms deviation of the alpha carbon atoms of 0.1 Å. In addition to the Ca^2^^+^ and Mg^2^^+^ ion binding sites seen in the wild-type structure there is the additional Mg^2^^+^ ion binding site as observed in the LLY^lec^Y62H-Le^y^ crystal structure. In the structure of LLY^lec^wt-Le^b^ there is one hydrogen bond from the hydroxyl group of Tyr62 to the *N*-acetyl moiety of Le^b^ and a second one via a water molecule to the hydroxyl group of Fuc 1 (**Figure 4C**). In the LLY^lec^Y62H-Le^b^ structure (**Figure 4D**) the bond from Tyr62 to the *N*-acetyl is replaced by a bifurcated hydrogen bond from His62 to the hydroxyl group of the Glc*N*Ac ring and the hydroxyl of the *N*-acetyl moiety off the ring. Overall, there was a net loss of a potential hydrogen bond as the water-mediated hydrogen bond between Tyr62 and Fuc 1 seen in the LLY^lec^wt-Le^b^ structure has been lost in LLY^lec^H62-Le^b^. The water molecules between the protein and Le^b^ are conserved in wild-type and mutant structures. LLY^lec^Y62H forms the same 23 van der Waals interactions with Le^b^ as does the wild-type protein: 15 with Fuc 1, 1 with Gal, 2 with Glc*N*Ac, and 4 with Fuc 2. The average B-factor of Le^b^ is 43.8 Å^2^ in LLY^lec^Y62H-Le^b^ (overall B-factor of protein complex is 23 Å^2^) which is very similar to 36.7 Å^2^ observed in the wild-type complex (overall B-factor of protein complex is 18 Å^2^). Thus, overall, there is a loss of one potential hydrogen bond between Le^b^ and mutant compared to wild-type.

Superposition of the crystal structures of LLY^lec^Y62H-Le^b^ and LLY^lec^Y62H-Le^y^ results in a rms deviation on the alpha carbons atoms of 0.1 Å. Fuc 1 and the Gal moieties of the Le^y^ and Le^b^ antigens superimpose quite well whereas Glc*N*Ac and Fuc 2 are shifted. Glc*N*Ac in Le^b^ moves 0.6 Å toward the protein due to a water-mediated hydrogen bond between the oxygen of the Glc*N*Ac-CH_2_OH group to the backbone oxygen of His62. Likewise, Fuc 2 is shifted by about 0.6 Å because the same interaction is not present in the Le^y^ complex.

### MOLECULAR SIMULATIONS OF LEWIS ANTIGEN INTERACTIONS

A total of 30,000 trajectory frames (100 × 30 ns) were collected for each Lewis antigen in complex with either LLY^lec^wt or the LLY^lec^Y62H. In the wild-type simulations, the Le^y^ antigen resided within the binding pocket more than the Le^b^ antigen (**Table 3**). However, when Y62 was mutated to a His this trend was reversed, with Le^b^ residing in the binding pocket more than Le^y^. These results are reflective of the SPR binding data, in that LLY^lec^wt has a higher affinity for Le^y^ over Le^b^, whereas LLY^lec^Y62H has a higher affinity for Le^b^ over for Le^y^.

**Table 3:** Cumulative totals of bound Lewis antigen over the course of 100 independent, 30 ns simulations starting from the Lewis bound conformation.

	wt	LLY^lec^Y62H
Le^y^	17,260 (57.5%)	13,835 (46.1%)
Le^b^	14,824 (49.4%)	16,080 (53.6%)

*All binding events within 3.5 Å rms deviation of the original antigen bound conformation were considered “bound.”*

**FIGURE 5:**
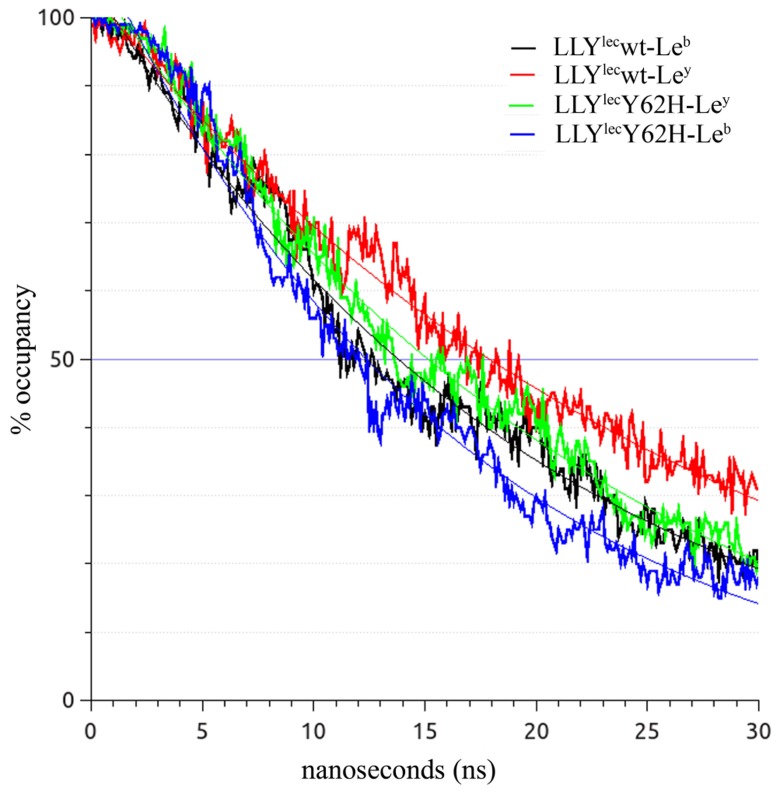
**Molecular dynamics modeling of the association of Lewis antigens with the LLY^lec^ binding sites**. Traces are averaged for all 100 simulations. Overall, the Lewis antigens occupied the binding site of either the wild-type or LLY^lec^Y62H less as the simulation progressed. However there are numerous areas of re-association, resulting in a spike in occupancy.

Lewis antigen dissociation rates were plotted as a function of occupancy (defined as <3.5 Å deviation from the original binding site) for all 100 simulations (**Figure 5**). This indicated a rapid dissociation rate which supports the SPR data where the on and off rates were too fast to be calculated. There is an overall trend for the antigens to occupy the binding site less as the simulations progressed. However, it is important to note that the curves are not purely exponential and contain distinct areas of peaks and troughs. This is indicative of the ligands moving rapidly in and out of the binding site of both LLY^lec^wt and LLY^lec^Y62H.

The simulation data reiterates the SPR finding that mutation of LLY^lec^Tyr62 to His62 has changed the binding specificity from a preference for Le^y^ by wild-type to a specificity for Le^b^ by the LLY^lec^Y62H mutant.

## DISCUSSION

The high level of expression of Le^y^ on the surface of epithelial tumor cells and low level expression elsewhere makes cell-bound Le^y^ a highly attractive target for anti-tumor agent delivery. In addition, the rigid nature and distinctive structure of Le^y^ lends itself to the ready development of highly specific protein recognition domains, whether in antibodies or other glycan-binding proteins.

Previously, we published the crystal structures of LLY^lec^wt in complex with the Le^b^ and Le^y^ antigens (Feil et al., 2012). These structures, together with computational modeling, were used to design and construct a glycan-binding domain with altered specificity for the Lewis antigens. The LLY^lec^Y62H mutation was suggested due to its structural similarity of His to Tyr and the potential to create a new interaction with Le^y^.

Surface plasmon resonance binding studies showed that LLY^lec^wt has a higher affinity for the Le^y^ antigen over Le^b^. This relationship was reversed for the designed LLY^lec^Y62H mutant, i.e., the Le^b^ antigen had higher affinity than the Le^y^ antigen. These results were unexpected, as the molecular modeling predicted that mutation of Tyr62 to His should decrease the number of hydrogen bonds with Le^b^ and increase the number of potential hydrogen bonds with Le^y^. The expectation was that the mutation would lead to an increase in affinity for Le^y^ and decrease in affinity for Le^b^, but in practice we found the reverse to be true.

To experimentally determine the interactions formed in the binding site as a result of the LLY^lec^Y62H mutation, we solved the crystal structures of LLY^lec^Y62H in complex with the Lewis antigens. In the LLY^lec^Y62H-Le^y^ complex, there was no additional bond formation between the Lewis antigen and His62, only a shift of a water molecule toward the His, as compared to its position relative to Tyr62 in the wild-type (**Figures 4A,B**). In the Le^b^ complex there was a net loss of a potential hydrogen bond to His62 (**Figures 4C,D**). In summary, the crystal structures did not show the bonding as predicted from the *in silico *mutant model (**Figure 2**) but revealed a net decrease in hydrogen bonding interactions to Le^b^ and no net change in bonding interactions with Le^y^. However, the SPR data revealed that the mutant had an increased affinity for Le^b^ and a decreased affinity for Le^y^.

Crystal structures are a static average snapshot of what happens in solution, so we used molecular dynamics studies to simulate the lectin domain-Lewis antigen binding mechanism in solution. In these simulations it was observed that the amino acid residues around the Lewis antigen binding site are mobile, particularly Tyr62. Thus, the inferred hydrogen bonding interactions between protein and ligands observed in the crystal structures may not necessarily be persistent in solution. This may help explain the unexpected consequences of our mutation.

The molecular dynamics studies provided us with complementary results to the SPR data: both approaches showed that the association and dissociation rates with Lewis antigens are extremely rapid, and that the relative affinities between antigens and wild-type or LLY^lec^Y62H domains are generally weak. The SPR data showed the strongest interaction being that of LLY^lec^wt to Le^y^ with a *K*_D_ of 78 μM and the weakest between LLY^lec^wt and Le^b^ with a *K*_D_ of 234.4 μM. Relative affinities of the wild-type or LLY^lec^Y62H binding sites for antigens was measured in the simulations by calculating how much time the Lewis antigens spent in the binding pocket. These dynamics studies agreed with the general trend of the biological results, suggesting that there was a reversal of Lewis antigen affinity when Tyr62 was mutated to a His. Interestingly, and again in concurrence with SPR data, the dynamics studies also suggest that the Lewis antigens bind weakly and transiently to the LLY^lec^ domain.

A recent example of protein engineering to alter Lewis antigen specificity is that of Norovirus virion protein 1 (NoV VP1), which specifically binds Le^b^ (Kubota et al., 2012). However, NoV VP1 has a deeper binding site with more extensive contacts to the Lewis antigen structure than seen in LLY^lec^. A single residue (Gln) in the Le^b^ binding site was mutated (to Asn) with the aim of increasing the affinity of the protein for Le^b^. The mutation increased the width of the binding site, allowing bonding between both fucose rings and amino acid side-chains. As expected, the mutant protein displayed a higher affinity for Le^b^.

In contrast, designing a high affinity Le^y^-specific LLY^lec^ mutant is more challenging: the crystal structures show that the Lewis antigens sit in a shallow hydrophobic pocket and that the only hydrogen bonds are between the basic residues His85, Arg112, and Arg120, and the α1-2 linked fucose. Few contacts are made between LLY^lec^ residues and other carbohydrate components of the Le^y^ and Le^b^ antigens (Feil et al., 2012). In addition, the structural differences between the Le^y^ and Le^b^ antigens are minor. These characteristics outline the challenges involved in redesigning LLY^lec^ in order to increase its specificity for Le^y^. Nevertheless, the results described herein show that, despite the strong structural similarities between Lewis antigens Le^y^ and Le^b^ and the shallow binding pocket, it is possible to alter the substrate specificity of the LLY lectin domain.

It has previously been demonstrated that LLY^lec^wt domain binds fucose alone (Farrand et al., 2008; Feil et al., 2012) and that the presence of LLY^lec^ enhances the pore-forming activity of LLY on platelets. Fucose is a commonly expressed carbohydrate, terminally decorating cell surface glycoproteins, including CD59 (Rudd et al., 1997; Wheeler et al., 2002), which is also the receptor for LLY (Wickham et al., 2011). This suggests the function of the lectin domain is perhaps as a “capture mechanism,” slowing down the passing monomers and increasing the local concentration of LLY in the microenvironment of the cell surface. A localized increase in LLY concentration would facilitate the oligomerization of LLY into prepores, prior to pore formation and consequent cell lysis. In this scenario, weak binding affinities for the fucose moieties, as demonstrated by SPR, would be beneficial as the monomers would need to be readily released for incorporation into assembling prepore oligomers. Further studies are required to confirm this mechanism.

## Conflict of Interest Statement

The authors declare that the research was conducted in the absence of any commercial or financial relationships that could be construed as a potential conflict of interest.

## Footnotes

1 http://www.pymol.org/

2 http://chemaxon.com/

3 www.biologic.com.au
